# Editorial^☆^

**DOI:** 10.1016/j.abrep.2025.100631

**Published:** 2025-09-12

**Authors:** Silvia Casale, Silke M. Müller, Matthias Brand

**Affiliations:** a Department of Health Sciences, University of Florence, Italy; b University of Duisburg-Essen, Germany

## 1 Introduction

The field of addictive behaviors has been increasingly challenged by the emergence of non-substance-related compulsive behaviors. While it is now widely recognized that excessive engagement in non-substance-related behaviors can be associated with negative mental health outcomes, a number of controversial issues remain—both cross-cutting and specific—calling for critical reflection.

This special issue aimed to provide a forum for theoretical reflection that addresses controversies and emerging topics in the field of behavioral addictions. We aim to stimulate and help readers identify directions for future research and clinical practice that are not only promising but also challenging. One overarching question of this special issue is: What are specific topics in the field of behavioral addiction research that warrant deeper investigation and new resolutions? While not intended as an exhaustive overview of all current debates, this collection aims to shed light on particularly pressing and thought-provoking issues.

### 1.1 Overview of content

This special issue brings together contributions that span almost the entire spectrum of behavioral addictions by focusing on specific forms – or those that are discussed as such –, such as gaming disorder or problematic social media use, while also including articles that address more general and cross-cutting controversies in the field. Three major themes are represented in this special issue: (1) diagnostic and conceptual controversies in identifying and assessing behavioral addictions, with proposals aimed at addressing these challenges; (2) therapeutic considerations relevant to treatment planning and clinical intervention; and (3) emerging issues that warrant further examination within conceptual and assessment frameworks. The special issue also includes evidence derived from an overview of systematic reviews and a meta-analysis.

#### 1.1.1 Diagnostic and conceptual controversies

Moretta and Wegmann examine the ongoing controversies surrounding the classification of problematic social media use (PSMU) and propose diagnostic criteria for this rapidly growing public health concern. By integrating a bottom-up process into a top-down one, they propose that PSMU should be diagnosed based on common criteria for addictive behaviors, such as gaming disorder (according to DSM-5 or ICD-11) and affective and cognitive symptoms potentially unique to PSMU, i.e. the preference for online social interaction and social-media specific fear of missing out. Given the central role of the reward system in addictive behaviors, they also review existing evidence and reflect on media-specific characteristics of social media platforms that may engage reward-related mechanisms.

Santoro and colleagues present a psychodynamic perspective on the mechanisms underlying behavioral addictions, proposing that these stem from childhood trauma. The authors suggest that traumatic experiences may activate dissociative processes that push painful mental states out of conscious awareness. Thus, addictive behaviors may function as emotional coping strategies to deal with these painful mental states, offering a false sense of control while ultimately intensifying inner fragmentation and discontinuities in self-experience.

Loscalzo and Giannini critically review the use of the addiction framework for defining (new) repetitive behaviors by stressing the risk of overpathologizing behaviors using a-priori and confirmative approaches. On the other hand, there might be a risk of not investigating potential new clinically relevant conditions. They also discuss the problem of lack of consensus regarding definitions of specific phenomena that are clinically relevant and close to addiction, such as obsessive–compulsive and related disorders. The authors claim thoroughly to investigate potential new clinical conditions in an unbiased manner, on the one hand, to avoid creating unnecessary new diagnoses, and on the other hand, to avoid missing any current issues relevant to public health.

#### 1.1.2 Guidelines for clinical intervention

The manuscript by Astrid Müller and colleagues highlights that psychotherapy research for Compulsive Buying-Shopping Disorder (CBSD) remains underdeveloped. This paper underscores the limitations of existing studies and proposes directions for future development, including assessment of psychotherapists' adherence to therapy manuals for cognitive-behavioral treatments, the efficacy evaluation of psychodynamic approaches, the inclusion of environmental and technological factors that may contribute to addictive use of online shopping platforms, and the inclusion of mental comorbidities. Moreover, the authors suggest adopting an experimental medicine framework that employs mechanism-focused methods to test the relevance of proposed interventions believed to underlie behavioral change.

Engel and colleagues propose a set of minimum requirements for evaluating treatment efficacy in studies on Compulsive Sexual Behavior Disorder (CSBD). These include measures of CSB symptom severity, behavioral engagement in CSB, and assessment of mechanisms of change. They emphasize the use of clinical interviews, and identify the prefrontal cortex and reward system as promising neural correlates for assessing treatment effects.

#### 1.1.3 Emerging issues

Montag and Elhai reflect on how Artificial Intelligence (AI) may contribute to the development of PSMU by offering users personalized feeds specifically designed to increase the time they spend online. They present the results of an empirical study which reveals that, especially among males, positive attitudes toward AI are associated with higher levels of PSMU, and highlight that time spent on social media has a mediating role in this relationship. Although preliminary for their cross-sectional nature, these findings suggest that continuing this line of research may be worthwhile, particularly by investigating the potential interactions between attitudes toward AI and well-established risk factors for PSMU.

Lo Coco and Rogers propose a model of the maintenance and exacerbation of body image and eating concerns in the context of PSMU (with a focus on social networks more specifically). They suggest that PSMU—whether generalized across multiple platforms or concentrated on a single one—may exacerbate and maintain body image and eating concerns. This occurs through appearance-driven engagement with social networks, appearance-focused online interactions, and additional putative moderators. The model also considers specific dimensions of PSMU that may contribute to these concerns.

Flayelle and colleagues call for research into the potential role of game design features in contributing to dysregulated gaming patterns. They propose that future research should investigate why specific video game design features may contribute to diminished self-regulation in gaming, when these features exert the strongest influence on the development of dysregulated gaming behaviors, and for whom they have the greatest impact. They also discuss briefly how these design features may be understood in the context of established theoretical frameworks for gaming disorder and provide examples of appropriate study designs that could effectively guide empirical research to support progress in research on the effects of design features in the context of addictive behaviors.

Finally, Kuss proposes a critical reflection on the intersection of technology, society, mental health, and public policy. She examines how the ubiquity of modern technologies and the pervasiveness of social media shape human behavior and identity, while acknowledging the adverse effects of excessive use—particularly in terms of psychological well-being. She proposes a public health lens, calling for a collaborative response from multiple stakeholders: governments, educators, parents, industry, and healthcare services.

#### 1.1.4 Evidence from systematic reviews and meta-analyses

By reviewing thirty-five systematic reviews and meta-analyses on psychological risk factors for PSMU, Fioravanti and colleagues identify both general predispositions shared with other forms of problematic Internet use and behavior-specific factors unique to PSMU. Notably, their findings align with the work of Moretta and Wegmann, not only in highlighting the presence of both common and specific factors, but also in identifying a preference for online social interaction as a distinctive feature of PSMU. More broadly, both contributions underscore commonalities and overlapping risk factors with other problematic online behaviors, as well as the role of specific motives and expectations in driving problematic engagement with social media platforms.

Focusing on a key criterion for differentiating between normative and problematic behaviors, An-Pyng Sun and colleagues present meta-analytic findings on both the dimensional and categorical stability of problematic gaming and gaming disorder. They emphasize that, while the categorical stability of gaming disorder may be less robust than that observed in major psychiatric disorders, it is nonetheless comparable to the stability found in gambling disorder. Regarding dimensional stability, they highlight that the severity of problematic gaming symptoms at baseline serves as a significant predictor of symptom severity at follow-up. This suggests a continuity of symptom intensity over time, reinforcing the conceptualization of problematic gaming as a condition with persistent characteristics.

### 1.2 Concluding remarks and future directions

One of the most persistent debates concerns the definition and identification of core symptoms in behavioral addictions, especially when these behaviors are inherently linked to Internet use. While scientific consensus has undoubtedly grown regarding which symptoms should be considered central—such as the impaired control over the behavior—and which are peripheral—such as tolerance—the efforts to ensure that this awareness is reflected in appropriate measurement instruments remain limited. With few exceptions, research continues to rely heavily on tools and (outdated) theoretical components that tend to conflate core and peripheral symptoms, perpetuating conceptual and diagnostic ambiguity.

Another recurring issue concerns terminological inconsistency—and, more importantly, conceptual conflation—in studies on problematic use of social media platforms. Researchers frequently formulate research hypotheses based on this conflation, without considering that not all social media platforms function as or are used as social networks. This reflects a broader tendency to overlook whether participants actually engage with social media platforms in socially networked ways which in turn hinders the clarification of whether we are dealing with two distinct phenomena: one potentially linked to the need for belonging and related expectations (i.e., problematic social network use), and another more rooted in the need to be visible, recognized, and admired (i.e., problematic social media use). An additional point is that a notable disconnect emerges between research on the effects of social media exposure on body image and research on the risk factors and consequences of problematic use — despite the fact that most widely used social platforms are predominantly appearance-focused. Both these issues may partly stem from the historical trajectory of research on social media use, which emerged during the rise of Facebook—a platform that most clearly embodied the features of a social network. As such, early studies naturally framed problematic use within the context of relational and networked interactions. However, as the digital landscape has diversified, maintaining this framework risks obscuring distinct patterns of use—some centered on connection, others on visibility and self-presentation.

Within but also beyond social networks, the addictive potential of online services appears to grow, not least through AI and the possibilities for individualizing content and offerings. Historically, specific providers offered specific services, but these are now increasingly merging. Applications are designed to keep users engaged for as long as possible, which leads to the integration of potentially addictive activities, e.g., the integration of gambling elements within computer games or purchasing functions within social media services. Given the similarities in affective and cognitive mechanisms in addiction development, one might ask the critical question of whether it is necessary to distinguish between different online behaviors at all, or whether everything is simply “problematic internet use.” Assuming we consider this differentiation to be important, the next question is whether differentiation is even possible (anymore) using current methods. Theoretically, uncontrolled surfing on the internet to satisfy a need to belong through online social interaction may be psychologically very different from the (uncontrolled) urge to buy products, and yet in practice both can be carried out on the same platform. The question of the (problematic) use of certain platforms alone may therefore not be sufficient in the future to adequately identify problematic behaviors. Only through thorough exploration of the potentially problematic behaviors and underlying mechanisms can effective measures for intervention or prevention be derived.

One challenge for future research that needs further dialogue is to systematically combine the most recent developments in theory building, etiology, empirical evidence about underlying psychological and neurobiological mechanisms and phenotypes of behavioral addictions, classification/nosology, diagnostic requirements and assessments as well as current knowledge about treatment efficacy and prevention. At international conferences and in journals with articles and commentaries on articles, such dialogues have started. We hope that this special issue on controversies in behavioral addiction research may stimulate further reflection and dialogue.

